# Pheochromocytomas and Paragangliomas as Causes of Endocrine Hypertension

**DOI:** 10.3389/fendo.2019.00333

**Published:** 2019-06-04

**Authors:** Letizia Canu, Gabriele Parenti, Giuseppina De Filpo, Massimo Mannelli

**Affiliations:** ^1^Department Experimental and Clinical Biomedical Sciences, University of Florence, Florence, Italy; ^2^Azienda Ospedaliero-Universitaria Careggi, Florence, Italy

**Keywords:** pheochromocytoma, paraganglioma, endocrine hypertension, chromaffin tumors, genetic-disease susceptibility

## Abstract

Chromaffin tumors are included among the causes of secondary hypertension because of the release of catecholamines. Nevertheless, the clinical, cardiovascular, and hypertensive picture of patients affected by pheochromocytomas/paragangliomas (PPGL) is extremely variable, due to the different quantitative and qualitative releasing activity of these tumors. A consistent percentage of these patients, about 20%, is normotensive and not affected by the characteristic symptomatic crises due to sudden release of catecholamines. The factors causing such wide clinical variability are many and probably not all known. It is well known that many of these tumors are genetically determined and that the genetic profile influences the biochemical characteristics and the biology of the tumors as well as the clinical presentation of the affected patients. The number of asymptomatic or poorly symptomatic patients is increased after the introduction of genetic screening and the early diagnosis in mutation carriers. In this paper we can review the genotype-phenotype correlation of PPGLs with a focus on the cardiovascular picture.

## Introduction

Pheochromocytomas and Paragangliomas (PPGL) are included, together with Primary Aldosteronism and Cushing's Syndrome, among the main causes of endocrine hypertension in view of their catecholamine releasing property (1). They are in fact tumors composed by neural crest derived cells that during their development acquire and maintain a sympatho-adrenergic phenotype characterized by the enzymatic and storage machineries present in the mature chromaffin cells (2). Nevertheless, at variance with the chromaffin cells of the normal adrenal medulla whose secreting activity is finely regulated by cholinergic sympathetic neurons, tumor chromaffin cells are not controlled in their discharging activity. Moreover, they sometimes display an incomplete enzymatic pathway causing a variable biochemical secretory pattern which may also include the co-release of peptides (3) differently active at the cardiovascular level.

These characteristics explain why PPGL show an extremely variable clinical pattern with very different blood pressure profiles (4, 5).

## Pheochromocytoma and Paraganglioma Clinical Picture

The variability of PPGL clinical presentation is since long well known (6) and underlined by the definition of “the great mimic” assigned to PPGL since the beginning of their discovery.

The increase in blood pressure (BP) is caused by the actions of tumor catecholamines on the adrenergic receptors. Activation of vascular α1 receptors causes peripheral vasoconstriction and an increase in vascular resistance. Activation of cardiac β1 receptors causes a chronotropic and inotropic effect on the myocardium thus leading to an increased output. Finally, high plasma concentrations of norepinephrine may act on the β1 receptors of the juxta-glomerular cells in the kidney, causing an activation of the renin-angiotensin-aldosterone system. As a whole, the tumor catecholamine discharge determines an increase in BP.

This increase may differ in many aspects, depending on many factors. Among these, the pattern of catecholamine release (continuous or sporadic), the amount of catecholamine release (determined mainly by the size of the PPGL), the type of catecholamine released (adrenaline, noradrenaline or dopamine), the possible co-release of peptides with different actions on vascular resistance (Table 1).

**Table 1:** Peptides possibly secreted by chromaffin tumors influencing blood pressure.

**Peptide**	**Effect**
Neuropeptide Y	Vasoconstriction
Renin	Hypertension
Endothelin	Vasoconstriction
ACE (Angiotensin Converting Enzyme)	Hypertension
ANP (Atrial Natriuretic Peptide)	Polyuria, hypotension
CGRP (Calcitonin G Related Peptide)	Vasodilation
PACAP (Pituitary Adenylate-cyclase activating peptide)	Vasodilation
Adrenomedullin	Vasodilation

Hypertension may present as continuous or intermittent, with or without sudden spikes, associated or not to other symptoms of adrenergic activation such as palpitations, sweating, trembling, anxiety.

The hypertensive crises can be so severe to be associated to cardiovascular complications such as myocardial ischemia, arrhythmias, heart failure, cerebral hemorrhage, sudden death.

**Figure 1:**
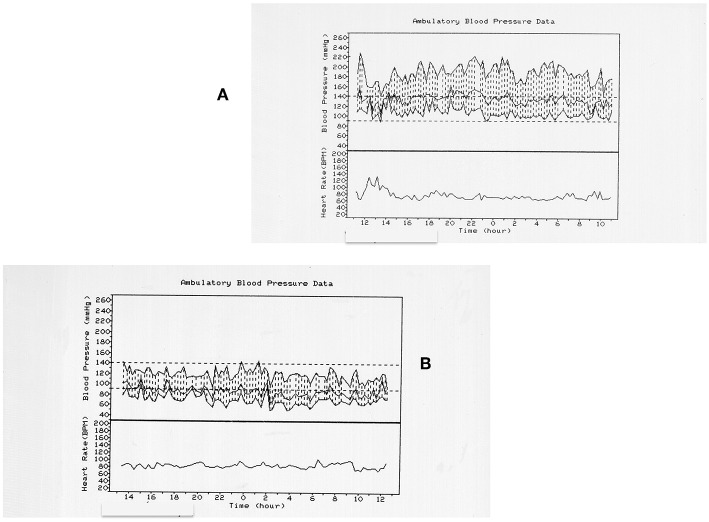
Twenty four hours blood pressure (BP) profiles in two different patients with pheochromocytoma. **(A)** refers to a 72 years old women with a right pheochromocytoma. The patient is hypertensive with BP spurts. **(B)** refers to a 79 years old man with a right pheochromocytoma. The patient is normotensive. The only common feature is the non-dipping profile of both the patients.

In a retrospective collaborative study conducted on behalf of the Italian Endocrine Society on 284 patients affected by a PPGL, only 60% presented hypertensive crises, about 20% presented a hypertension not different from that of patients with essential hypertension, and 21% resulted normotensive (5) (Figure 1).

The secretory pattern, continuous or intermittent, can also influence the arterial vasoconstriction modifying the vascular responsiveness to catecholamines. In fact, a continuous exposure to high concentrations of catecholamines causes a down regulation of adrenergic receptors (7), thus counteracting the increase in vascular resistance.

In the very rare occurrence of a PPGL in pregnancy, the clinical presentation is influenced by the physiological cardiovascular changes occurring in this condition such as the decrease in vascular resistance and the expansion of circulating volume. In pregnancy, the PPGL-induced hypertension if often erroneously diagnosed as pre-eclampsia with deleterious consequences on the mother and the fetus (8).

The surgical removal of the tumor normalizes the catecholamine plasma levels and abolishes the risks of acute cardiovascular complications but not always is able to normalize BP in hypertensive patients (9).

## Genetics

In the last 20 years the spectrum of the PPGL susceptibility genes has progressively enlarged so that at present about 35–40% of PPGL is caused by a germ-line mutation of one of these genes (10, 11) (Table 2). The genetic profiling of PPGL has shown the occurrence of two main clusters characterized by the activation of two different pathogenic pathways (12). Cluster 1 includes mainly tumors linked to mutations of *VHL* (von Hippel-Lindau) and *SDHx* (succinate-dehydrogenase) genes and characterized by the induction of a pseudohypoxia mechanism. Cluster 2 includes tumors mainly linked to mutations of *NF1* (neurofibromatosis type 1) and *RET* (responsible for the occurrence of Multiple Endocrine Neoplasia type 2) genes and characterized by the activation of tyrosine-kynase pathway.

**Table 2:** Genes implied in the pathogenesis of Pheochromocytoma/paraganglioma.

**Gene**		**Frequency of mutation**	**Mutation type**
ATRX	ATRX, chromatin remodeler	< 5%	S
BRAF	B-Raf proto-oncogene, serine/threonine kinase	< 2%	S
CDKN2A	Cyclin dependent kinase inhibitor 2A	< 2%	S
EGLN1/PHD2	egl-9 family hypoxia inducible factor 1	< 1%	G/S
EPAS1	Endothelial PAS domain protein 1	6–12%	M/S
FGFR1	Fibroblast growth factor receptor 1	~1%	S
FH	Fumarate hydratase	1–2%	G
H3F3A	H3 histone family member 3A	< 2%	M
HRAS	HRas proto-oncogene, GTPase	7–8%	S
IDH2	Isocitrate dehydrogenase [NADP(+)] 2, mitochondrial	< 0.5%	S
KIF1B	Kinesin family member 1B	< 5%	G/S
KMT2D	Lysine methyltransferase 2D	< 2%	G/S
MAX	MYC associated factor X	1–2%	G/S
MDH2	Malate dehydrogenase 2	< 2%	G
MERTK	MER proto-oncogene, tyrosine kinase	< 2%	G
MET	MET proto-oncogene, receptor tyrosine kinase	< 2% o < 2–10%	G/S
NF1	Neurofibromin 1	3% o 20–25%	G/S
RET	Ret proto-oncogene	5–6%	G/S
SDHA	Succinate dehydrogenase complex flavoprotein subunit A	< 1%	G/S
SDHAF2	Succinate dehydrogenase complex assembly factor 2	< 1%	G
SDHB	Succinate dehydrogenase complex iron sulfur subunit B	8–10%	G
SDHC	Succinate dehydrogenase complex subunit C	1–2%	G
SDHD	Succinate dehydrogenase complex subunit D	5–7%	G
TMEM127	Transmembrane protein 127	1–2%	G
TP53	Tumor protein p53	< 5%	S
VHL	von Hippel-Lindau tumor suppressor	7–10%	G/S

*G, germline mutation; S, somatic mutation; M, mosaicism. Modified by NGS in PPGL (NGSnPPGL) Study Group et al. (11)*.

The two different pathogenic pathways cause different PPGL phenotypes in terms of secretory pattern and biochemistry. It has demonstrated that VHL and MEN2 PPGL are indeed different (13).

In VHL syndrome, PPGL present a lower expression of TH (tyrosine hydroxylase), the rate limiting enzyme in catecholamine biosynthesis and an almost absent activity of the PNMT (phenylethanolamine-N–methyltransferase), responsible for the conversion of norepinephrine to epinephrine. In fact, in VHL tumors, the PNMT gene has been found hypermethylated and therefore downregulated (14). As a consequence, VHL PPGL have a lower total tissue content of catecholamines, represented by norepinephrine while MEN2 PPGL have higher tissue content of catecholamines and release both epinephrine and norepinephrine (15). These biochemical differences explain why MEN2 patients result more symptomatic and have a higher incidence of hypertension, mainly paroxysmal in comparison with VHL patients.

Moreover, cluster 1 is characterized by a more immature phenotype, especially in SDHB related PPGL. These tumors, which display the higher occurrence of metastatic forms, not only do not express PNMT but often also lack dopamine-β-hydroxylase activity (the enzyme responsible for the conversion of dopamine to norepinephrine) (16). Therefore, many SDHB related PPGL release dopamine which, acting at the vascular and renal DA1 receptors causes vasodilation and natriuresis, respectively, thus counteracting the hypertensive effects of norepinephrine.

Many other factors might contribute to a different BP profile. Succinate, which is increased in SDHx mutated PPGL, causes an increase of plasma renin activity in rats (17) but circulating succinate does not differ between hypertensive patients and normotensive controls (18).

Finally, the familial genetic screening permits the discovery of mutation carriers and the early diagnosis of small PPGL whose scanty releasing activity does not cause hypertension.

## Therapy

The therapy of PPGL is surgical and in the sporadic forms, the removal of the tumor leads to the disease cure in a very high percentage of cases. Nevertheless, a pre-surgical medical therapy is generally recommended (19) and the drugs of choice are α-blockers. The two most often used drugs are phenoxybenzamine (PBZ) and doxazosine (DXZ). PBZ is a non-selective (blocks both α1 and α2 receptors), non-competitive drug while DXZ is a selective (blocks only α1 receptors), competitive (can be displaced from the receptors by endogenous norepinephrine) drug. The α2-receptor blocking activity of PBZ exerted at the presynaptic level causes an increased discharge of norepinephrine from the sympathetic terminals so that almost always it causes a painful tachycardia which needs the addition of a β blocker.

It is worth mentioning that, in the absence of α-blocking therapy, β-blockers are contraindicated in patients with PPGL. In fact, inhibiting the vasodilation mediated by vascular β2-receptors, they can worsen a supervening catecholamine-induced hypertensive crisis.

The α blocking action causes a vasodilation, a reversal of the receptor dawn-regulation and a decrease in BP. This latter causes in turn an increase in blood volume and all these actions not only limit the pre-surgical hypertensive spikes but also avoid the dangerous hypotensive post-surgical crises.

According to the Endocrine Society guidelines (20), a target BP of < 130/80 mmHg while seated and >90 mm Hg systolic while standing seems reasonable, with a target heart rate of 60–70 bpm seated and 70–80 bpm standing. If this target is not achieved by the use of only α blockers, other hypotensive drugs such as calcium channel blockers, angiotensin converting enzyme inhibitors or angiotensin II receptors antagonists can be added in the pre-surgical treatment.

## Conclusions

PPGL are among the causes of endocrine hypertension. Nevertheless, at variance with the other causes, the BP pattern they present is extremely variable, being the hypertension often intermittent or paroxysmal and even absent in about 20% of cases.

## Author Contributions

MM, LC, GD, and GP contributed conception of the paper. LC wrote the first draft of the manuscript. GD and GP wrote sections of the manuscript. MM revised the manuscript. All authors read and approved the submitted version.

### Conflict of Interest Statement

The authors declare that the research was conducted in the absence of any commercial or financial relationships that could be construed as a potential conflict of interest. The handling Editor declared a past collaboration with one of the authors LC.
